# Genetically Predicted Circulating Concentrations of Micronutrients and Risk of Amyotrophic Lateral Sclerosis: A Mendelian Randomization Study

**DOI:** 10.3389/fgene.2021.811699

**Published:** 2022-01-17

**Authors:** Changqing Mu, Yating Zhao, Chen Han, Dandan Tian, Na Guo, Chenguang Zhang, Ruixia Zhu, Xiaoqian Zhang, Jian Zhang, Xu Liu

**Affiliations:** ^1^ Department of Neurology, First Affiliated Hospital of China Medical University, Shenyang, China; ^2^ Department of Cell Biology, Key Laboratory of Cell Biology, Ministry of Public Health, Shenyang, China; ^3^ Key Laboratory of Medical Cell Biology, Ministry of Education, China Medical University, Shenyang, China

**Keywords:** amyotrophic lateral sclerosis, micronutrient, mendelian randomization study, genome-wide association study, susceptibility

## Abstract

Amyotrophic lateral sclerosis (ALS) is a progressive and devastating neurodegenerative disease with increasing incidence and high mortality, resulting in a considerable socio-economic burden. Till now, plenty of studies have explored the potential relationship between circulating levels of various micronutrients and ALS risk. However, the observations remain equivocal and controversial. Thus, we conducted a two-sample Mendelian randomization (MR) study to investigate the causality between circulating concentrations of 9 micronutrients, including retinol, folate acid, vitamin B12, B6 and C, calcium, copper, zinc as well as magnesium, and ALS susceptibility. In our analysis, several single nucleotide polymorphisms were collected as instrumental variables from large-scale genome-wide association studies of these 9 micronutrients. Then, inverse variance weighted (IVW) approach as well as alternative MR-Egger regression, weighted median and MR-pleiotropy residual sum and outlier (MR-PRESSO) analyses were performed to evaluate causal estimates. The results from IVW analysis showed that there was no causal relationship of 9 micronutrients with ALS risk. Meanwhile, the three complementary approaches obtained similar results. Thus, our findings indicated that supplementation of these 9 micronutrients may not play a clinically effective role in preventing the occurrence of ALS.

## Introduction

Amyotrophic lateral sclerosis (ALS) is a fatal heterogeneous neurodegenerative disorder which selectively damages upper and lower motor neurons (van Es et al., 2017). It is typically characterized by progressive motor deficits such as dysphagia, dysarthria, muscle atrophy and weakness of the trunk and extremities, and even respiratory failure (Masrori and Van Damme, 2020). Globally, the number of individuals suffered from ALS reached 222,801 in 2015 and it is expected to constantly increase, reaching 376,674 by 2040 (Arthur et al., 2016). Moreover, the per capita cost related to ALS was the highest among various neurological diseases, and in the United States alone, the standardized total expense of ALS was estimated to range from $279 to $472 million (Gladman and Zinman, 2015). Due to severe clinical symptoms and huge socio-economic burden, an increasing number of studies have been implemented to investigate the predisposition factors of ALS and explore its possible pathogenesis.

In recent years, micronutrients including vitamins and minerals were found to be related to multiple neurodegenerative diseases such as AD and PD (Hu et al., 2013; Boulos et al., 2019). Regarding ALS, the relationship between various micronutrients and ALS risk has not yet been determined. For example, a center-based survey including 202 ALS patients and 208 healthy controls showed that lower circulating levels of vitamin C and higher levels of retinol were significantly related to an increased risk of ALS (Wang et al., 2020). Moreover, Peters et al. observed a significant association between circulating zinc levels and ALS susceptibility based on a prospective cohort study involving 520,000 European participants (Peters et al., 2021). However, some other evidence indicated no statistically significant association between circulating levels of vitamin C as well as retinol and ALS risk (Iwasaki et al., 1995; Paraskevas et al., 1997). Similarly, a case-control study by Forte et al. failed to observe a difference in circulating zinc levels between ALS cases and healthy controls (Forte et al., 2017). Due to the small sample size, inevitable potential confounding factors and reverse causation, the results from the observational studies mentioned above are inconsistent.

For overcoming the conventional bias of observational studies, mendelian randomization (MR) analysis could reveal the causal relationship between predisposition factors and ALS susceptibility by applying single nucleotide polymorphisms (SNPs) as instrumental variables (IVs) as well as large-scale data from genome-wide association studies (GWASs). Indeed, three previous MR studies have revealed no causal effect of serum 25-hydroxyvitamin D, iron and selenium on ALS risk (Larsson and Roos, 2020; Cai et al., 2021; He and Cui, 2021). However, up till now, apart from these three micronutrients, no systematic MR analysis has been published for other vitamins and minerals. Therefore, we conducted this two-sample MR analysis to more accurately infer the causal relationship between circulating levels of various micronutrients and ALS risk.

## Materials and Methods

### Study Design, Instruments Selection and Data Sources

**FIGURE 1:**
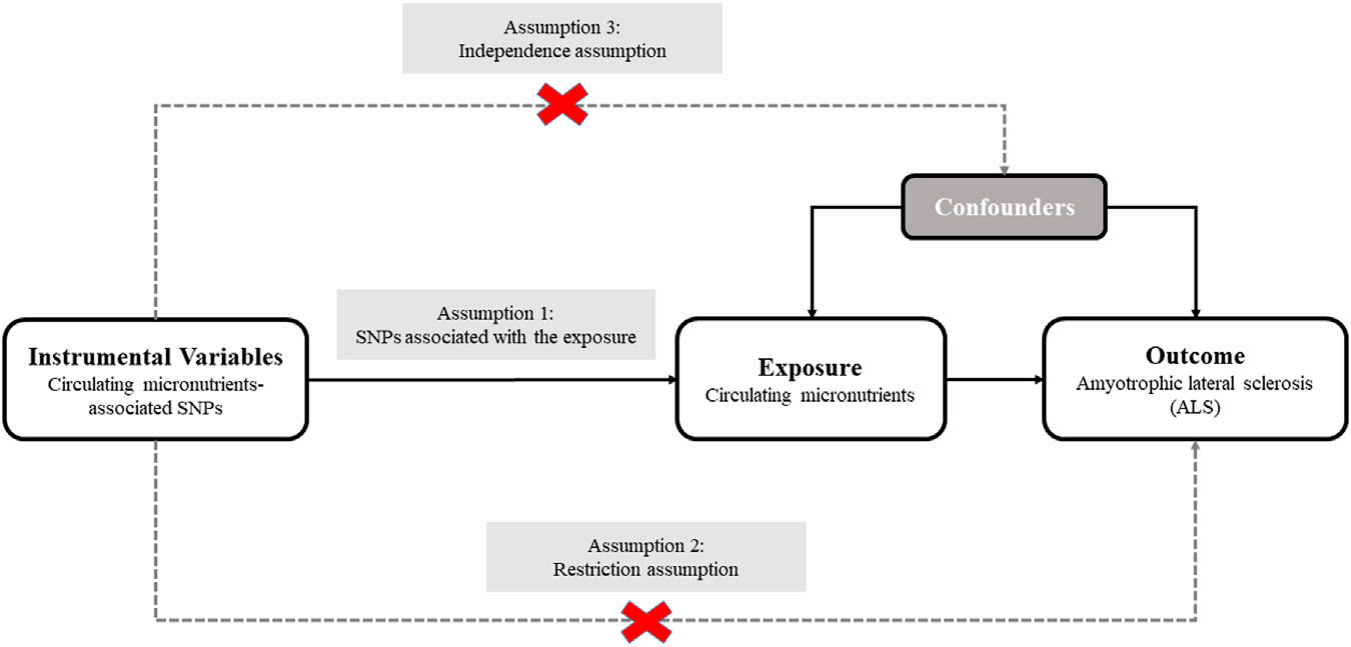
The overall design of Mendelian randomization analysis in the present study.

We designed a two-sample MR analysis to investigate the causal relationship of plasma micronutrients on ALS (Pierce and Burgess, 2013). The complete two-sample design of our MR framework is displayed in Figure 1. For determining interesting exposures, a search for the published GWASs on the circulating levels of various micronutrients was conducted using PubMed databases. A total of 7 GWASs involving 9 micronutrients were identified, including 5 vitamins (retinol, folate acid, vitamin B12, B6 and C) and 4 minerals (calcium, copper, zinc and magnesium) (Hazra et al., 2009; Meyer et al., 2010; Mondul et al., 2011; Evans et al., 2013; Grarup et al., 2013; O'Seaghdha et al., 2013; Zheng et al., 2021). The detailed information of GWASs related to 9 exposures are listed in Table 1. Next, the candidate SNPs representing the circulating levels of 9 micronutrients were selected by a genome-wide significance threshold (*p* < 5.00E-08). Lastly, we used a *r* ^*2*^ threshold <0.01 to prune all selected SNPs, which retaining 43 SNPs with the lowest *p*-value for detecting relationship between circulating levels of 9 micronutrients and ALS risk. Regarding GWAS dataset for ALS susceptibility, summary association statistics was collected from the most recent and comprehensive GWAS, which totally included 80,610 individuals of European descent (20,806 ALS cases and 59,804 controls) (Nicolas et al., 2018). For these 43 selected SNPs, β coefficients and standard errors for effect of SNPs on 9 micronutrients and ALS risk were extracted from abovementioned GWAS datasets for subsequent analysis (Supplementary Table S1). Since we only retrieved GWAS summary-level data rather than individual-level data, no additional ethical permit was required.

**TABLE 1:** Summary of details on GWASs and related datasets involving nine micronutrients in our study.

Exposures	Cohorts or datasets	Participants	Publicly available websites	PMID
Vitamin C	Fenland study, EPIC InterAct study, EPIC Norfolk study, EPIC-CVD study	52,018 individuals of European ancestry	https://doi.org/10.6084/m9.figshare.13227443.v1	33203707
Vitamin B12	Icelanders, Danish-Inter99, Danish-Health2006	45,576 individuals of European ancestry	NA	23754956
Folate acid	Icelanders, Danish-Inter99, Danish-Health2006	37,341 individuals of European ancestry	NA	23754956
Retinol	ATBC study, PLCO study, NHS studies, InCHIANTI	8,902 individuals (mostly European ancestry)	NA	21878437
Vitamin B6	CGEMS study, SHARe study	4,763 individuals of European ancestry	NA	19744961
Calcium	AGES, ARIC study, BLSA, CoLAUS, CROATIA-Vis, CROATIA-Korcula, CROATIA-Split, FHS, HABC, InCHIANTI, LBC 1936, LOLIPOP EW A, LOLIPOP EW P, LOLIPOP EW610, OGP Talana, ORCADES, SHIP, RS, CHS	39,400 individuals of European ancestry	NA	24068962
Magnesium	ARIC study, FHS, RS	15,366 individuals of European ancestry	NA	20700443
Copper	QIMR studies	2,603 individuals of European ancestry	https://genepi.qimr.edu.au/general/downloadable.cgi	23720494
Zinc	QIMR studies	2,603 individuals of European ancestry	https://genepi.qimr.edu.au/general/downloadable.cgi	23720494

EPIC, European Prospective Investigation into Cancer and Nutrition; CVD, cardiovascular disease; ATBC, the alpha-tocopherol, beta-carotene cancer prevention study; PLCO, Prostate, lung, colorectal, and ovarian cancer screening trial; NHS, the nurses’ health study; InCHIANTI, the invecchiare in chianti Study; CGEMS, the cancer genetic markers of susceptibility project; SHARe, the SNP Health Association Resource; AGES, age gene/environment susceptibility reykjavik study; ARIC study, the atherosclerosis risk in communities study; BLSA, Baltimore Longitudinal Study of Aging; CoLAUS, Cohorte Lausannoise; FHS, Framingham Heart Study; HABC, The Health, aging and body composition; LBC1936, lothian birth cohort 1936; LOLIPOP, London Life Sciences Population study; EW, European whites; OGP, Ogliastra Genetic Park; ORCADES, Orkney Complex Disease Study; SHIP, Study of Health in Pomerania; RS, The Rotterdam Study; CHS, The Cardiovascular Health Study; QIMR, the queensland institute of medical research; NA, not available.

### Statistical Analyses

In order to investigate the potential causal relationship between circulating levels of 9 micronutrients and ALS risk, two-sample MR analysis was performed by employing five statistical methods including Wald method, inverse-variance weighted (IVW), MR-Egger, weighted median and MR-Pleiotropy Residual Sum and Outlier (MR-PRESSO). In detail, we applied Wald method to evaluate the causal relationship for the micronutrient with only one related SNP as IV. If more than one SNP involved, IVW method was implemented as the primary analysis to explore the causal effect on ALS risk. Moreover, the Cochran’s *Q* and funnel plot test was used to evaluate the heterogeneity of the causal estimates among multiple SNPs. Subsequent leave-one-out sensitivity analysis was performed to investigate whether a single SNP was driving the IVW point estimate.

Additionally, in the IVW MR analysis, the ratios of SNP-micronutrients to SNP-ALS were combined in a meta-analysis to explore the causal relationship between circulating levels of micronutrients and ALS risk (Burgess et al., 2013). The premise of IVW analysis is that all SNPs are valid IVs, and it could lead to an increased statistical power and accurate estimate when the core assumption of MR is satisfied. However, if the genetic variants affect ALS susceptibility through other pathways than micronutrients (horizontal pleiotropy), the causal estimate may be biased (Hemani et al., 2018). Therefore, we conducted a complementary analysis by using the weighted median and MR-Egger and MR-PRESSO methods. Specifically, weighted median method could maintain reliable estimates even if up to half of IVs are invalid (Bowden et al., 2016). Regarding MR-Egger regression method, it does not limit the slope to pass zero in the micronutrients–ALS causal estimate and identifies the presence of potential pleiotropy by calculating the intercept value (Bowden et al., 2015). Besides, the MR-PRESSO global and outlier test was also implemented as an approach for detecting pleiotropic outliers (Verbanck et al., 2018).

Given 9 exposures involved in our MR analysis, we set the statistically significant threshold of *p*-value to 5.56E-03 (0.05/9) according to Bonferroni correction. Meanwhile, a *p*-value, which implied the suggestive evidence for a possible causal role, was set to range from 5.56E-03 to 0.05. All above analyses were performed using R version 4.0.3 software with R-packages (MendelianRandomization, TwoSampleMR and MR-PRESSO).

## Results

### Circulating Levels of Vitamins and ALS Risk

**FIGURE 2:**
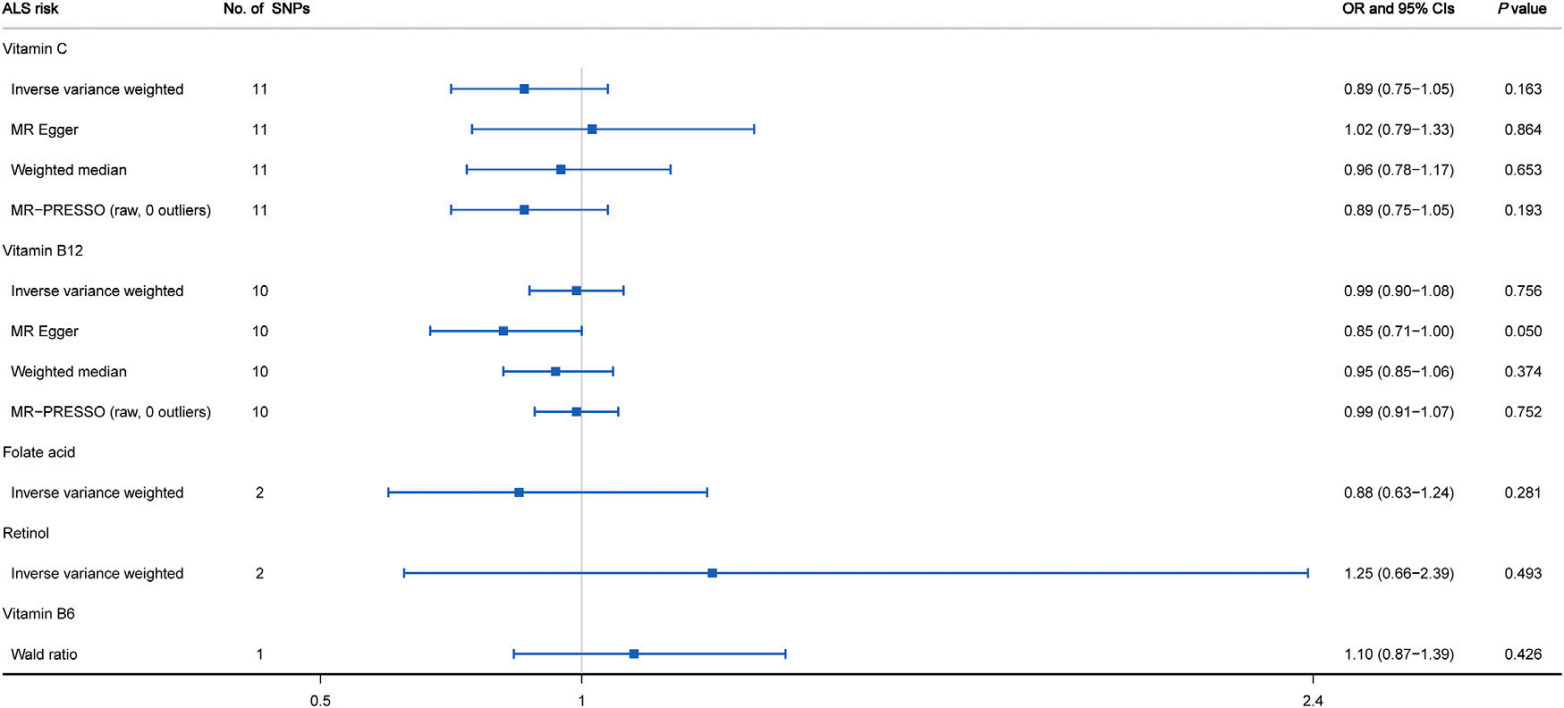
MR analysis of genetically predicted levels of circulating vitamins and ALS risk.

As shown in Figure 2 and Supplementary Figure S1, the MR estimates obtained by the IVW method suggested that predicted circulating concentrations of four vitamins including vitamin C (OR = 0.89, 95% CI: 0.75 to 1.05, *p* = 0.163), vitamin B12 (OR = 0.99, 95% CI: 0.90 to 1.08, *p* = 0.756), folate acid (OR = 0.88, 95% CI: 0.63 to 1.24, *p* = 0.281) and retinol (OR = 1.25, 95% CI: 0.66 to 2.39, *p* = 0.493) were causally unrelated to ALS susceptibility. In addition, for each vitamin, we did not observe any obvious heterogeneity through the Cochran’s *Q* test (*p* > 0.05). However, regarding vitamin C, the visual inspection of the funnel plot showed slight asymmetry and potential heterogeneity (Supplementary Figure S2). When leave-one-out sensitivity analysis was performed, the change of IVW point estimate was detected for vitamin C when omitting rs2559850, but not for vitamin B12 (Supplementary Figure S3). Moreover, 3 additional alternative approaches were also applied containing MR-Egger, weighted median and MR-PRESSO. The results further supported that there was no causal relationship between vitamin C as well as vitamin B12 and ALS risk (Figure 2). Based on the MR-Egger regression analysis, we did not observe evidence of potential pleiotropy between circulating levels of vitamin C and ALS risk (intercept = -0.012, *p* = 0.173). However, a mild pleiotropy could be detected for vitamin B12 (intercept = 0.024, *p* = 0.036). In addition, no potential SNP outliers were identified by MR-PRESSO test (vitamin C: *p* = 0.249; vitamin B12: *p* = 0.538). Lastly, regarding vitamin B6 with only one related SNP as IV, by using the Wald ratio method, we did not find any causal effect on ALS risk (OR = 1.10; 95% CI: 0.87–1.39; *p* = 0.426).

### Circulating Levels of Minerals and ALS Risk

**FIGURE 3:**
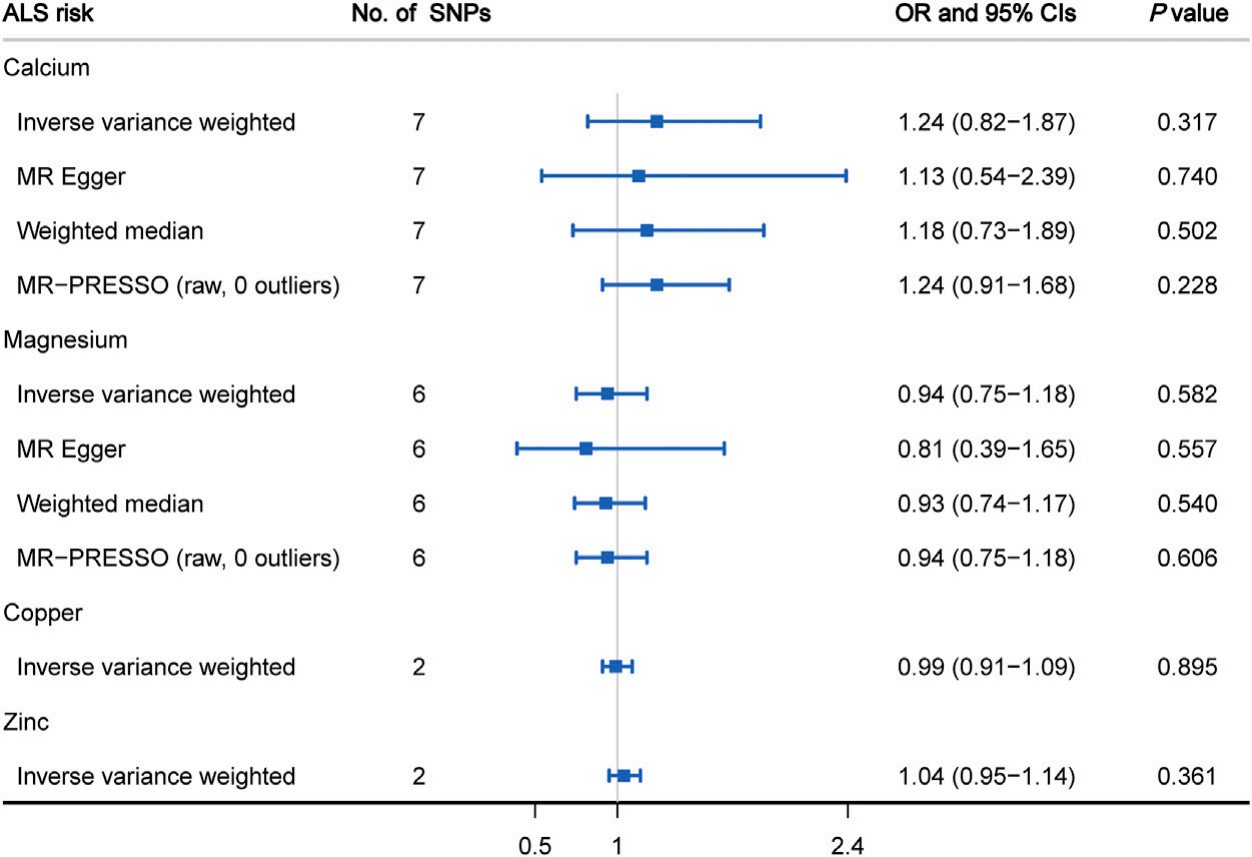
MR analysis of genetically predicted levels of circulating minerals and ALS risk.

Figure 3 displays the causal estimates of four minerals on ALS risk. In the primary analysis using IVW method, we did not observe any causal relationship between circulating levels of calcium (OR = 1.24, 95% CI: 0.82 to 1.87, *p* = 0.317), magnesium (OR = 0.94, 95% CI: 0.75 to 1.18, *p* = 0.582), copper (OR = 0.99, 95% CI: 0.91 to 1.09, *p* = 0.895), zinc (OR = 1.04, 95% CI: 0.95 to 1.14, *p* = 0.361) and ALS occurrence (Supplementary Figure S4). Meanwhile, there was no significant heterogeneity measured by Cochran’s *Q* test (*p* > 0.05) and funnel plot (Supplementary Figure S2). Subsequent sensitivity analysis involving calcium and magnesium showed that no single SNP dominated the IVW point estimate (Supplementary Figure S3). Regarding calcium and magnesium, the results in IVW analysis were further verified by using MR- Egger, weighted median and MR-PRESSO methods (Figure 3). Additionally, no significant pleiotropic effects for included SNPs were detected by MR-Egger regression test (calcium: intercept = 0.003, *p* = 0.789; magnesium: intercept = 0.011, *p* = 0.659). In the subsequent analysis using MR-PRESSO method, we did not find any outliers for these two minerals (calcium: *p* = 0.829; magnesium: *p* = 0.183).

## Discussion

In spite of the short survival time and the increased incidence of ALS, unfortunately, no effective preventions have been found so far (Traxinger et al., 2013; Xu et al., 2020). Thus, for further exploring possible preventive measures, MR analysis was introduced to investigate whether 9 micronutrients were causally related to ALS risk or not. Here, we did not find any causal relationship between 9 micronutrients and ALS susceptibility, including 5 vitamins (retinol, folate acid, vitamin B12, B6 and C) and 4 minerals (calcium, copper, zinc and magnesium).

Recently, an increasing number of studies showed that high homocysteine levels were closely associated with a variety of pathological processes, including apoptosis and autophagy, mitochondrial dysfunction and oxidative stress, thereby damaging motor neurons and leading to ALS occurrence (Zoccolella et al., 2010). Moreover, vitamin B6, B12 and folate acid would change homocysteine status by mediating one-carbon metabolism (Hao et al., 2007; Clare et al., 2019). Thus, a series of experimental studies have explored whether these three B vitamins can participate in ALS occurrence. For example, a cellular study by Hemendinger et al. (2011) reported vitamin B12 could block the apoptosis of motor neuron-like cells by reducing homocysteine levels while methylfolate could not. Meanwhile, in the SOD1G93A transgenic mouse model of ALS, folate acid alone or a combination of folate acid and vitamin B12 exhibited neuroprotective effects on motor neurons and retarded the onset of ALS (Zhang et al., 2008). More importantly, several observational studies have also been performed to investigate the association between B vitamins and ALS susceptibility. In 2007, Izumi et al. observed that the mean survival time of 18 patients in the vitamin B12 treatment group was significantly longer than that of 16 patients without any treatment (Izumi and Kaji, 2007). Conversely, a randomized controlled study including 373 Japanese ALS patients showed that intramuscular injection of high-dose vitamin B12 had no significant effect on the development of ALS (Kaji et al., 2019). Regarding vitamin C and retinol, as two of the most common antioxidants, they may have the potential to delay the initiation and progression of ALS by reducing oxidative stress (Esposito et al., 2002). In previous experiments on familial ALS model mice, no significant difference in the age of ALS onset was detected between the vitamin C treatment group and control group, while long-term supplementation of retinoic acid would shorten the lifespan of ALS mice (Nagano et al., 2003; Crochemore et al., 2009). However, a case-control study involving 40 ALS patients and 87 healthy controls did not support the association between circulating retinol levels and ALS risk (Molina et al., 1999). Additionally, in a meta-analysis of five large cohorts including 1,053,575 participants, Fitzgerald et al. reported that high dietary intake of vitamin C was not related to ALS susceptibility (Fitzgerald et al., 2013). Overall, the discrepant results of these studies on 5 vitamins aforementioned may be due to the small sample size, limited follow-up time, confounding and reverse causality. Thus, we introduced the more reliable MR analysis and finally inferred that there was no causal relationship between genetically predicted circulating levels of folate acid, vitamin B12, B6, and C, retinol and the risk of ALS.

Regarding the four minerals analyzed in this study, many observational studies focusing on their relationship with ALS susceptibility have been performed. However, until now, the results of these studies were inconsistent and inconclusive. For instance, in a case-control study involving 392 participants, Peters et al. (2016) observed the statistically significant association between copper and zinc and ALS susceptibility. Nevertheless, in other case-control studies, these association cannot be replicated (Roos et al., 2013; Forte et al., 2017). Thus, we have introduced the MR analysis, hoping to provide a broader and more reliable perspective on the causal relationship between 4 minerals and ALS risk. Here, our results did not support that genetically predicted circulating levels of calcium, copper, zinc and magnesium were causally related to the occurrence of ALS.

Several advantages of our study were as follows. First, this is the first MR analysis for determining the causal relationship between various micronutrients including 5 vitamins as well as 4 minerals and ALS risk. Second, we adopted large-scale GWAS datasets involving thousands of participants to ensure the robustness and authenticity of our MR analysis. Third, for all micronutrients, no heterogeneity was measured between SNPs by Cochran’s *Q* test. Moreover, we chose three complementary methods, including MR-Egger, weighted median and MR-PRESSO, which further confirmed most of the results from IVW analysis. Some limitations should be noted in the present MR analysis. First, a mild pleiotropy for vitamin B12 can be detected using MR-Egger method. Meanwhile, in the leave-one-out analysis for vitamin C, we found that omission of rs2559850 had slight influence on the causal relationship. Moreover, the funnel plot of vitamin C indicated potential heterogeneity. Thus, the note should be taken when interpreting the relationship of vitamin B12 and C with ALS risk. Second, almost all participants of GWAS datasets in our analysis were Europeans, which limited the general application in other populations. Third, since the data involving the clinical characteristics of ALS patients were not available, such as age, gender, age at onset and disease progression, etc., we can only analyze the potential association between micronutrients and ALS susceptibility.

## Conclusion

In conclusion, this MR study provided no evidence for causal association between genetically predicted circulating levels of 9 micronutrients and ALS risk. These findings indicate that supplementation of these 9 micronutrients might not be clinically helpful in preventing the occurrence of ALS.

## Data Availability

The datasets used and/or analyzed during the current study are available from the corresponding author on reasonable request.

## Supplementary Material

Supplementary Table 1 Characteristics of the SNPs associated with circulating micronutrients and their association with ALS.

Supplementary Figure 1 Forest plots of the causal effects of vitamins-associated SNPs on ALS risk. **(A)** vitamin C, **(B)** vitamin B12, **(C)** Folate acid, **(D)**: Retinol.

Supplementary Figure 2 Funnel plots of the causal effects of micronutrients-associated SNPs on ALS risk. **(A)** vitamin C, **(B)** vitamin B12, **(C)** Calcium, **(D)** Magnesium.

Supplementary Figure 3 Sensitivity analyses of the causal effects of micronutrients-associated SNPs on ALS risk. **(A)** vitamin C, **(B)** vitamin B12, **(C)** Calcium, **(D)** Magnesium.

Supplementary Figure 4 Forest plots of the causal effects of minerals-associated SNPs on ALS risk. **(A)** Calcium, **(B)** Magnesium, **(C)** Copper, **(D)** Zinc.
