# Supplementary figures and images for: Genetically Predicted Circulating Concentrations of Micronutrients and Risk of Amyotrophic Lateral Sclerosis: A Mendelian Randomization Study
Source: Front Genet. 2022 Jan 17;12:811699. doi: 10.3389/fgene.2021.811699 (PMC8801789; doi:10.3389/fgene.2021.811699)

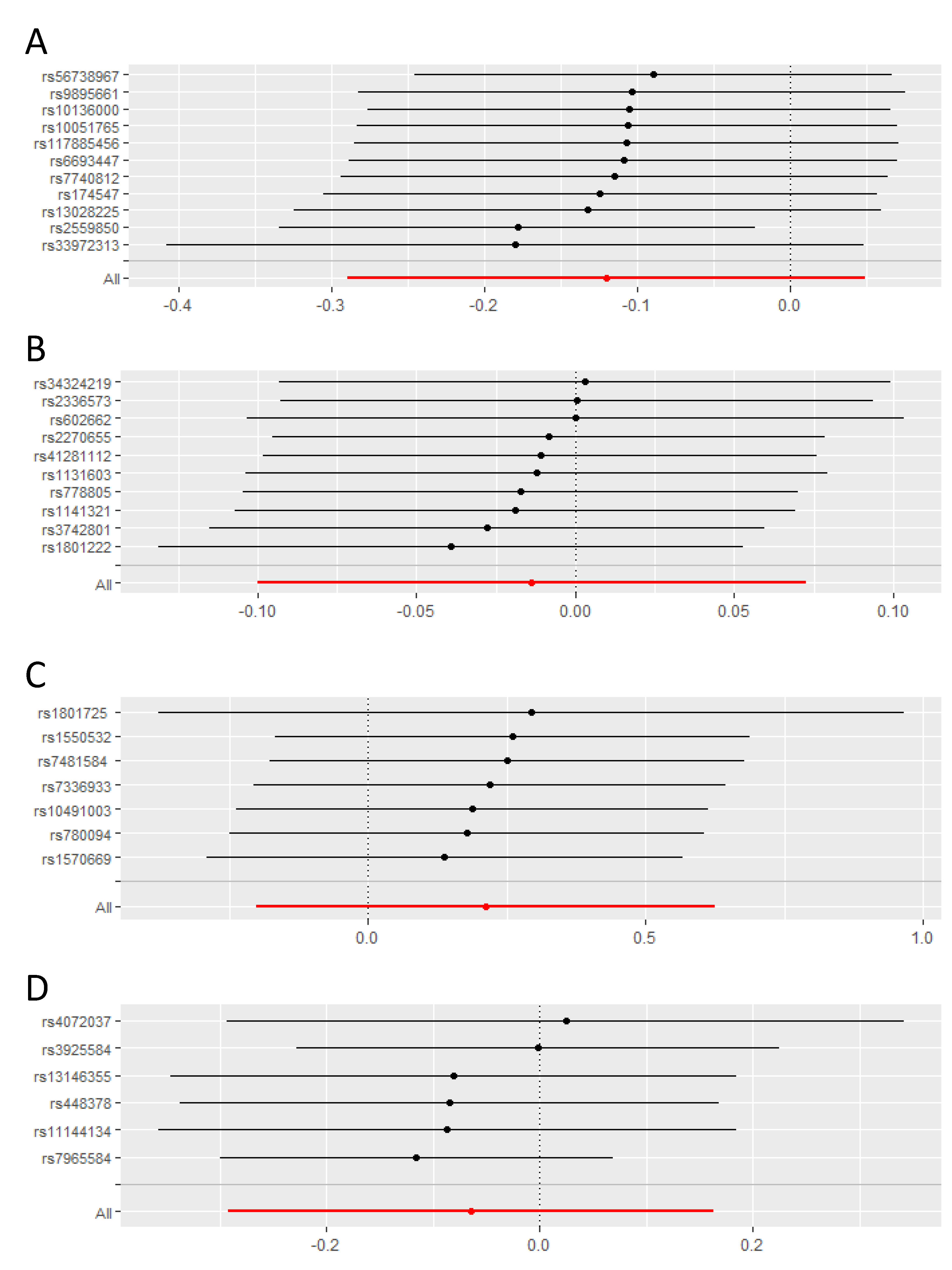

Supplement: Supplementary file 1 [file Image3.TIFF]

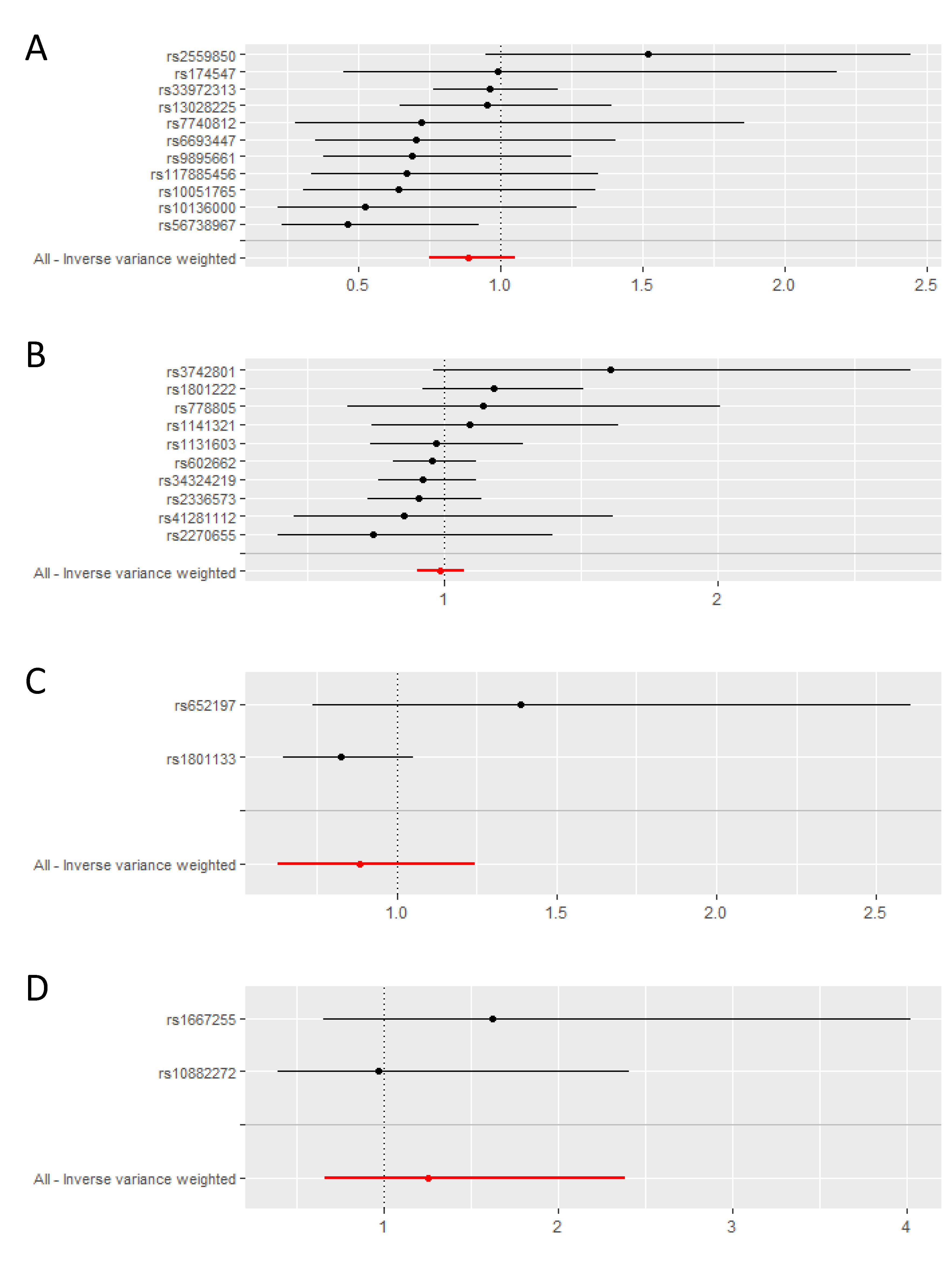

Supplement: Supplementary file 2 [file Image1.TIFF]

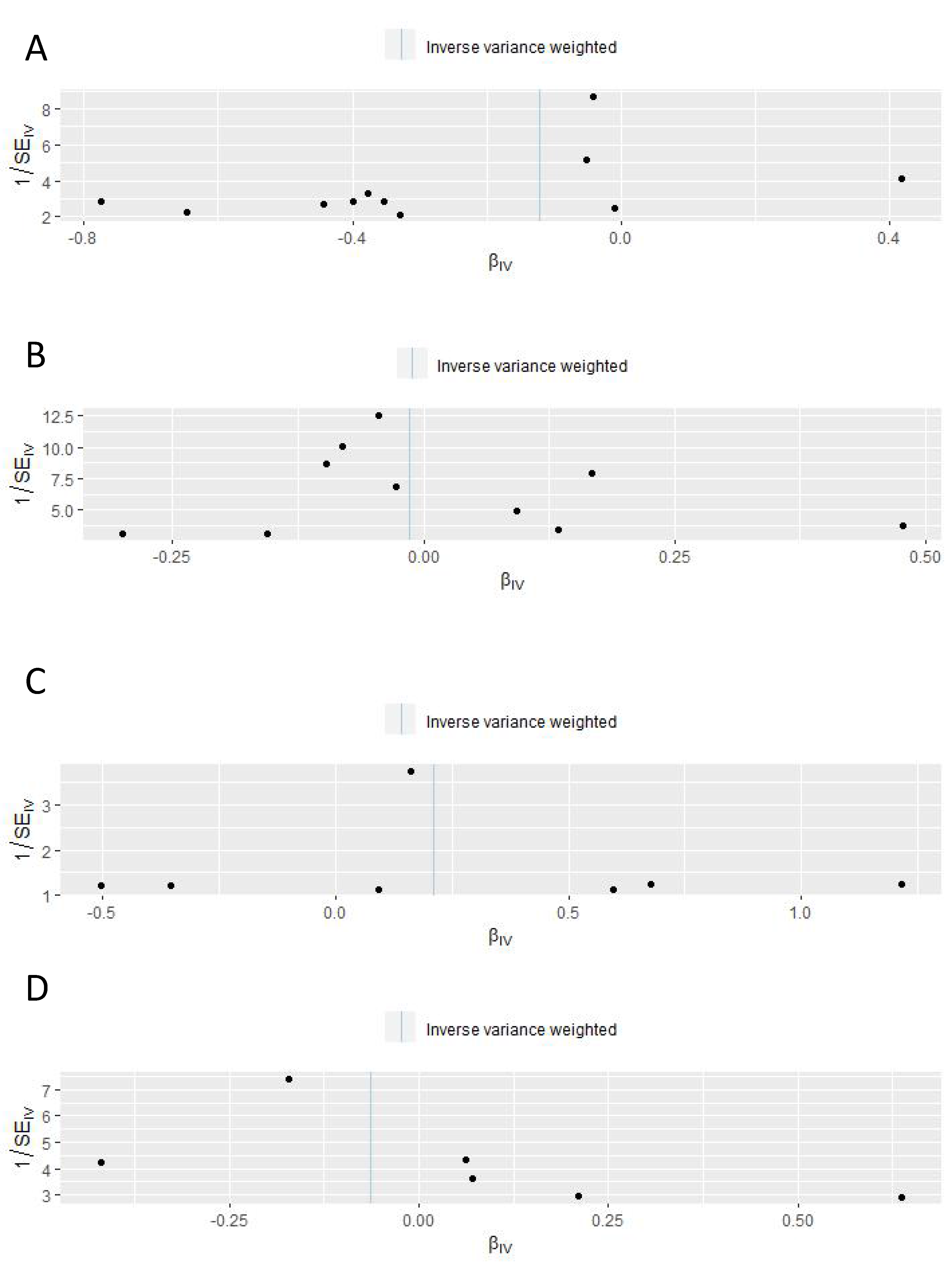

Supplement: Supplementary file 4 [file Image2.TIFF]

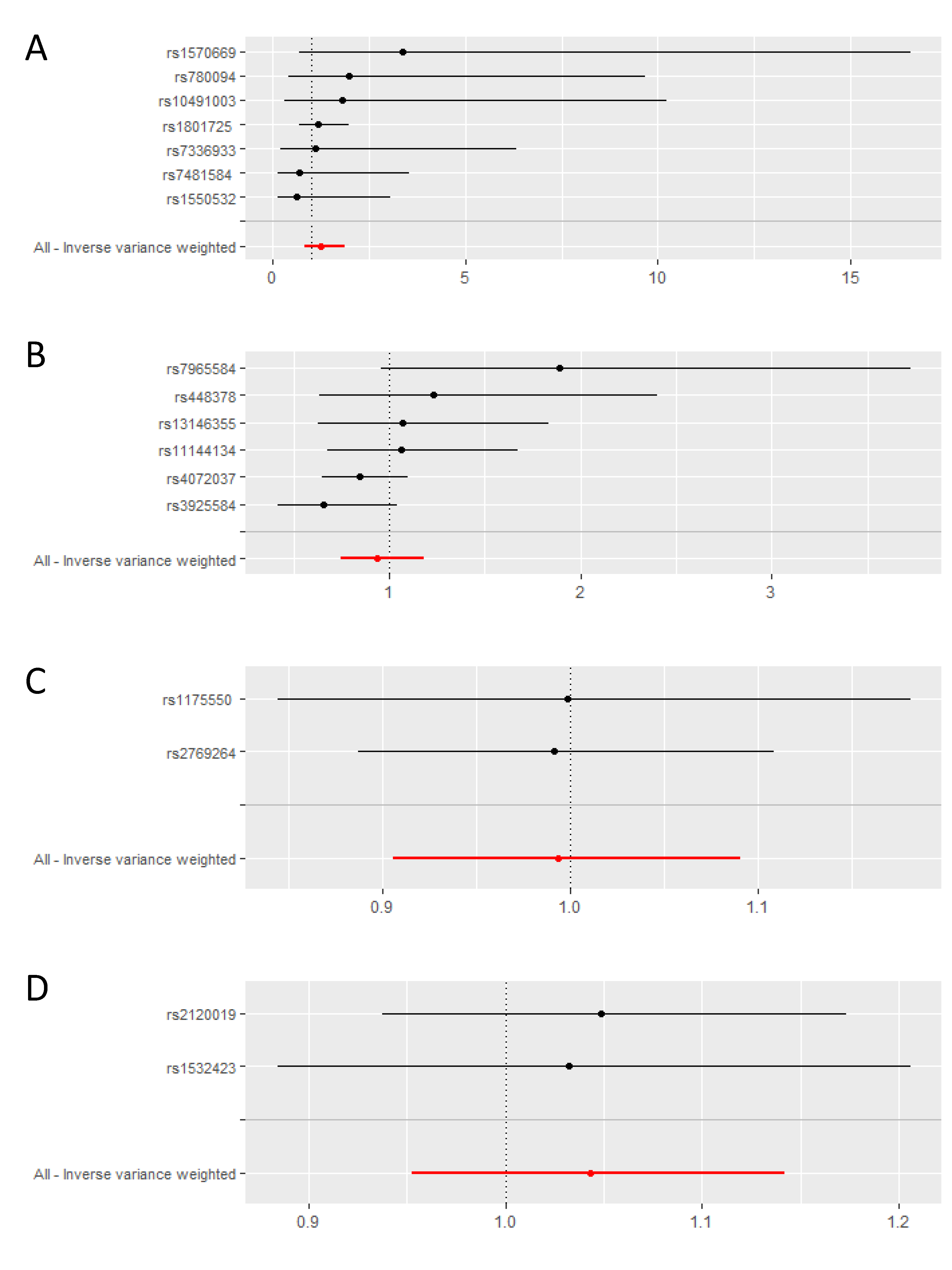

Supplement: Supplementary file 5 [file Image4.TIFF]
