# Supplementary material for: Genetically Predicted Circulating Concentrations of Micronutrients and Risk of Amyotrophic Lateral Sclerosis: A Mendelian Randomization Study
Source: Front Genet. 2022 Jan 17;12:811699. doi: 10.3389/fgene.2021.811699 (PMC8801789; doi:10.3389/fgene.2021.811699)
Supplement: Supplementary file 3 [file Table1.DOCX]

**Table S1. Characteristics of the SNPs associated with circulating micronutrients and their association with ALS.**

|  |  |  | **Micronutrients’ level** | | |  | **ALS** | | |
| --- | --- | --- | --- | --- | --- | --- | --- | --- | --- |
| **Micronutrients** | **SNP** | **EA** | **beta^*^** | **se** | ***P*** |  | **beta^†^** | **se** | ***P*** |
| Vitamin C | rs56738967 | C | 0.041 | 0.007 | 7.62E-10 |  | -0.0318 | 0.0146 | 0.0296 |
|  | rs2559850 | A | 0.058 | 0.006 | 6.30E-20 |  | 0.0243 | 0.014 | 0.08425 |
|  | rs174547 | C | 0.036 | 0.007 | 3.84E-08 |  | 3.00E-04 | 0.0145 | 0.9854 |
|  | rs13028225 | T | 0.102 | 0.009 | 2.38E-30 |  | -0.0052 | 0.0199 | 0.7938 |
|  | rs7740812 | G | 0.038 | 0.006 | 1.88E-09 |  | 0.0125 | 0.0184 | 0.4969 |
|  | rs6693447 | T | 0.039 | 0.006 | 6.25E-10 |  | -0.0138 | 0.0138 | 0.3155 |
|  | rs33972313 | C | 0.36 | 0.018 | 4.61E-90 |  | 0.0147 | 0.0414 | 0.7226 |
|  | rs10051765 | C | 0.039 | 0.007 | 3.64E-09 |  | 0.0173 | 0.0146 | 0.2382 |
|  | rs9895661 | T | 0.063 | 0.008 | 1.05E-14 |  | -0.0237 | 0.0192 | 0.2177 |
|  | rs10136000 | A | 0.04 | 0.007 | 1.33E-08 |  | -0.0259 | 0.0181 | 0.1515 |
|  | rs117885456 | A | 0.078 | 0.012 | 1.70E-11 |  | -0.0312 | 0.0277 | 0.2605 |
| Vitamin B12 | rs2270655 | G | 0.099 | 0.015 | 5.68E-12 |  | 0.0297 | 0.0321 | 0.3551 |
|  | rs3742801 | T | 0.053 | 0.007 | 2.28E-13 |  | 0.0253 | 0.014 | 0.07063 |
|  | rs1801222 | G | 0.119 | 0.007 | 7.24E-74 |  | -0.0199 | 0.0149 | 0.1827 |
|  | rs778805 | A | 0.05 | 0.008 | 1.04E-10 |  | 0.0067 | 0.0144 | 0.6418 |
|  | rs1141321 | C | 0.07 | 0.007 | 5.11E-25 |  | -0.0065 | 0.0143 | 0.6505 |
|  | rs1131603 | C | 0.222 | 0.015 | 2.11E-48 |  | 0.0062 | 0.0321 | 0.8467 |
|  | rs602662 | A | 0.171 | 0.007 | 8.15E-138 |  | -0.0077 | 0.0136 | 0.572 |
|  | rs34324219 | C | 0.235 | 0.011 | 2.54E-109 |  | 0.0191 | 0.0232 | 0.4098 |
|  | rs41281112 | C | 0.181 | 0.015 | 4.60E-34 |  | 0.0282 | 0.059 | 0.6329 |
|  | rs2336573 | T | 0.313 | 0.019 | 2.89E-60 |  | -0.0304 | 0.0361 | 0.4004 |
| Folate acid | rs652197 | C | 0.069 | 0.01 | 5.73E-13 |  | -0.0226 | 0.0222 | 0.3095 |
|  | rs1801133 | G | 0.114 | 0.008 | 6.65E-53 |  | 0.022 | 0.0142 | 0.1228 |
| Retinol | rs1667255 | C | 0.03 | 0.004 | 6.35E-14 |  | -0.0145 | 0.0139 | 0.2981 |
|  | rs10882272 | C | -0.03 | 0.004 | 6.51E-15 |  | -0.0011 | 0.014 | 0.9379 |
| Vitamin B6 | rs1256335 | G | -0.14 | 0.02 | 1.40E-15 |  | 0.0133 | 0.0167 | 0.4259 |
| Calcium | rs1550532 | C | 0.018 | 0.003 | 8.20E-11 |  | -0.0091 | 0.0149 | 0.5415 |
|  | rs10491003 | T | 0.027 | 0.005 | 4.80E-09 |  | 0.0161 | 0.0238 | 0.4984 |
|  | rs780094 | T | 0.017 | 0.003 | 1.30E-10 |  | 0.0115 | 0.0138 | 0.4042 |
|  | rs1801725 | T | 0.071 | 0.004 | 8.90E-86 |  | 0.0114 | 0.019 | 0.5511 |
|  | rs7481584 | A | -0.018 | 0.003 | 1.20E-10 |  | 0.0064 | 0.0149 | 0.6663 |
|  | rs7336933 | A | -0.022 | 0.004 | 9.10E-10 |  | -0.002 | 0.0197 | 0.9206 |
|  | rs1570669 | A | -0.018 | 0.003 | 9.10E-12 |  | -0.0219 | 0.0146 | 0.1334 |
| Magnesium | rs3925584 | C | -0.06 | 0.01 | 5.20E-16 |  | -0.0252 | 0.0141 | 0.07447 |
|  | rs4072037 | C | -0.1 | 0.01 | 2.01E-36 |  | -0.0172 | 0.0135 | 0.2044 |
|  | rs13146355 | G | -0.05 | 0.01 | 6.27E-13 |  | 0.0035 | 0.0137 | 0.8004 |
|  | rs11144134 | T | -0.11 | 0.01 | 8.21E-15 |  | -0.0068 | 0.0254 | 0.7878 |
|  | rs448378 | G | -0.04 | 0.01 | 1.25E-08 |  | 0.0084 | 0.0136 | 0.535 |
|  | rs7965584 | G | -0.07 | 0.01 | 1.05E-16 |  | 0.0446 | 0.0242 | 0.06461 |
| Copper | rs1175550 | A | -0.198 | 0.032 | 5.03E-10 |  | 3.00E-04 | 0.017 | 0.9855 |
|  | rs2769264 | G | 0.313 | 0.034 | 2.63E-20 |  | 0.0026 | 0.0178 | 0.8853 |
| Zinc | rs1532423 | A | 0.178 | 0.026 | 6.40E-12 |  | 0.0057 | 0.0141 | 0.6859 |
|  | rs2120019 | C | -0.287 | 0.033 | 1.55E-18 |  | 0.0137 | 0.0164 | 0.4037 |

SNP: single-nucleotide polymorphism; EA: effect allele; se: standard error; *The beta coefficients represent the change in circulating levels of micronutrients (in SD unit) for each additional effect allele; †The beta coefficients represent the log odds ratio of ALS for each additional effect allele.
